# Mechanisms and therapeutic significance of autophagy modulation by antipsychotic drugs

**DOI:** 10.15698/cst2018.11.161

**Published:** 2018-10-25

**Authors:** Ljubica Vucicevic, Maja Misirkic-Marjanovic, Ljubica Harhaji-Trajkovic, Nadja Maric, Vladimir Trajkovic

**Affiliations:** 1 Institute for Biological Research, University of Belgrade, Belgrade, Serbia.; 2 Clinic of Psychiatry, Clinical Centre of Serbia and School of Medicine, University of Belgrade, Belgrade, Serbia.; 3 Institute of Microbiology and Immunology, School of Medicine, University of Belgrade, Dr. Subotica 1, 11000 Belgrade, Serbia.

## Abstract

In this review we analyze the ability of antipsychotic medications to modulate macroautophagy, a process of controlled lysosomal digestion of cellular macromolecules and organelles. We focus on its molecular mechanisms, consequences for the function/survival of neuronal and other cells, and the contribution to the beneficial and side-effects of antipsychotics in the treatment of schizophrenia, neurodegeneration, and cancer. A wide range of antipsychotics was able to induce neuronal autophagy as a part of the adaptive stress response apparently independent of mammalian target of rapamycin and dopamine receptor blockade. Autophagy induction by antipsychotics could contribute to reducing neuronal dysfunction in schizophrenia, but also to the adverse effects associated with their long-term use, such as brain volume loss and weight gain. In neurodegenerative diseases, antipsychotic-stimulated autophagy might help to increase the clearance and reduce neurotoxicity of aggregated proteotoxins. However, the possibility that some antipsychotics might block autophagic flux and potentially contribute to proteotoxin-mediated neurodegeneration must be considered. Finally, the anticancer effects of autophagy induction by antipsychotics make plausible their repurposing as adjuncts to standard cancer therapy.

## INTRODUCTION

The term autophagy (‘self-eating’ in Greek) refers to a group of evolutionarily conserved homeostatic mechanisms employed by eukaryotic cells to eliminate aged, unused, and damaged cytoplasmic components through lysosomal degradation 1. Three main types of autophagy that differ in the mechanisms of delivery of autophagic substrates to lysosomes have been termed microautophagy, chaperone-mediated autophagy, and macroautophagy 2. Macroautophagy (hereafter autophagy), the best characterized form of autophagy in mammalian cells, relies on sequestration of autophagic cargo by double-membraned vesicles known as autophagosomes, which fuse with lysosomes to form autolysosomes 3. In addition to its physiological role in maintaining intracellular homeostasis, autophagy is an important part of the adaptive protective response against metabolic, hypoxic, oxidative, infectious, inflammatory, proteotoxic, and drug-induced stress 4. On the other hand, when extensive or activated inappropriately, autophagy can contribute to apoptotic/necrotic cell demise or function as an alternative programmed cell-death pathway 5.

Neurons, being post-mitotic, heavily rely on autophagic clearance of proteotoxins and damaged mitochondria, and are thus particularly vulnerable to autophagy disturbances 6,7. In addition to controlling neuronal survival and death during stress, autophagy has been shown to regulate presynaptic neurotransmission 8. Moreover, dysregulation of neuronal autophagy has recently been implicated in the pathogenesis of schizophrenia 9, a chronic mental disorder in which the abnormalities in social behavior and perception of reality are associated with altered neurotransmission 10. Antipsychotic drugs used to manage psychosis in schizophrenia mainly act by reducing dopaminergic neurotransmission 11. They could be broadly classified as typical and atypical, the latter being developed more recently, and possessing additional anti-serotonergic activity. However, the complexity and beneficial/adverse consequences of dopamine-dependent and -independent actions of antipsychotics on neurons and other cell types are far from being completely elucidated. More than three decades ago it has been reported that administration of now discontinued antipsychotic reserpine caused autophagy in rat liver 12. Many years later, a screening of 480 bioactive small molecules for autophagy-inducing capacity revealed that three among eight most efficient compounds were FDA-approved antipsychotic drugs trifluoperazine, fluspirilene, and pimozide 13. A number of studies subsequently confirmed the ability of various typical and atypical antipsychotic drugs to modulate autophagy in different cell types, including neurons.

In this concise review, we analyze autophagy regulation by antipsychotic medications, focusing on its molecular mechanisms and consequences for the neuronal function/survival and treatment of schizophrenia and neurodegeneration. The autophagy modulation by antipsychotics in other cell types and cancer is also briefly discussed.

## ANTIPSYCHOTIC-MEDIATED MODULATION OF NEURONAL/BRAIN AUTOPHAGY 

Autophagy induction is mainly assessed by measuring the conversion of autophagic protein microtubule-associated light chain 3 (LC3)-I to its lipidated, autophagosome-localized form LC3-II 14. However, as LC3-II is subsequently degraded in lysosomes, its increase can also result from autophagy inhibition 14. To avoid confusion due to inadequate interpretation of LC3 data, we have focused on the studies that assessed autophagic turnover (flux) and/or used pharmacological/genetic modulation of autophagy to confirm its biological effects 14. However, due to known technical difficulties with assessing autophagic flux *in vivo* 15, the available *in vivo* data are presented with the limitations explicitly noted.

### Activation of neuronal autophagy by antipsychotics

In most of the *in vitro* studies, antipsychotics stimulated autophagic flux in neuronal cells (**Table 1**), as confirmed by the increase in LC3 conversion in the presence of lysosomal inhibitors, appearance of autophagosomes and their fusion with lysosomes, and/or degradation of selective autophagic targets, such as autophagic cargo receptor sequestosome 1/p62. Pimozide, sertindole, olanzapine, and trifluoperazine induced autophagy in human neuronal cell line SH-SY5Y 16,17,18,19, while trifluoperazine, fluphenazine, and methotrimeprazine efficiently increased autophagic flux in human or rat primary neurons 20,21. A structure-activity study in rat striatal neurons revealed that an amino-containing substituent at the N10 position was important for LC3-II increase by tricyclic antipsychotics trifluoperazine, promazine, chlorpromazine, triflupromazine, thioridazine, and mesoridazine, although no flux data were provided 22. Pimozide and fluspirilene, having a biphenyl core and a 3-4 carbon linker to a tertiary amine, display structural similarities to tricyclics, which further emphasizes the importance of this structural scaffold in neuronal autophagy induction by antipsychotics 22. Risperidone and haloperidol increased the numbers of acidic, presumably autophagic vesicles, in the cytoplasm of SH-SY5Y cells 18, but their ability to stimulate autophagic flux was not directly confirmed. Importantly, antipsychotics were also able to increase autophagic markers in the brains of experimental animals *in vivo*. Pimozide, olanzapine, and clozapine increased LC3-II conversion, autophagosome numbers, autophagic degradation of p62, and/or expression of autophagy genes in mouse or rat frontal cortex and/or hippocampus 16,18,23, while trifluoperazine increased LC3 conversion in a zebrafish model of Parkinson’s disease caused by the deficiency of phosphatase and tensin homolog-induced kinase 1 19.

**Table 1:** Table 1. Modulation of neuronal/brain autophagy by antipsychotics. "Flux" refers to autophagic flux; ↑ denotes increase/activation; ↓ denotes decrease/inhibition; n.a. - not assessed; AMPK - AMP-activated protein kinase; ATG - autophagy related; DA - dopaminergic; PINK1 - Phosphatase and tensin homolog -induced kinase 1; ROS - reactive oxygen species; TDP43 - TAR DNA-binding protein 43; TFEB - transcription factor EB; ULK1 - Unc-51 like autophagy activating kinase.

**Agent**	**Cells/Tissues**	**LC3-II**	**Flux**	**Mechanisms**	**Effects**	**Ref.**
Pimozide	SH-SY5Y cells	↑	↑	AMPK/ULK1↑	Tau clearance; Neuronal spine density↑	16
Pimozide	Mouse hippocampus	↑	↑	AMPK/ULK1↑	Tau clearance; Memory improvement	16
Sertindole	SH-SY5Y cells	↑	↑	ROS↑	Cell death	17
Olanzapine	SH-SY5Y cells	↑	↑	ROS↑; ATG mRNA↑	Apoptotic death upon autophagy inhibition	18
Olanzapine	Mouse hippocampus and frontal cortex	↑	n.a.	ATG mRNA↑	Apoptotic death upon autophagy inhibition	18
Trifluoperazine	SH-SY5Y cells	↑	↑	TFEB↑; p62 mRNA↑	n.a.	19
Trifluoperazine	Deyolked zebrafish lysate	↑	↑	p62 mRNA↑	DA neurons rescue in PINK1^-/-^ mutants	19
Trifluoperazine	Human neurons *in vitro*	↑	↑	n.a.	α-synuclein clearance Neuronal survival↑	21
Fluphenazine	Rat neurons *in vitro*	↑	↑	n.a.	TDP43 clearance; Neuronal survival↑	20
Methotrimeprazine	Rat neurons *in vitro*	↑	↑	n.a.	TDP43 clearanceM Neuronal survival↑	20
Clozapine	Rat frontal cortex	↑	n.a.	AMPK/ULK1↑	n.a.	23
Clozapine	Rat neurons *in vitro*	↓	↓	Autophagosome-lysosome fusion↓	n.a.	24
Haloperidol	Rat neurons *in vitro*	↓	↓	Autophagosome-lysosome fusion↓	n.a.	24

### Inhibition of neuronal autophagy by antipsychotics

Similarly to the above findings, the *in vitro* treatment with haloperidol and clozapine increased LC3-II levels in rat primary neurons 24. However, this was attributed to a decrease in LC3-II degradation due to a block of autophagosome-lysosome fusion and subsequent inhibition of autophagic proteolysis 24. While these findings indicate a unique ability of haloperidol and clozapine to prevent autophagy completion, they also imply that the *in vivo* increase in steady-state LC3-II levels by clozapine 23 might reflect inhibition, rather than induction of autophagic flux. It is also possible that haloperidol and/or clozapine actually triggered an autophagic response, but later blocked it at the lysosomal stage. An assessment of the earlier stages of autophagy and autophagy-inducing signaling pathways, as well as recently developed methods for measuring neuronal autophagic flux *in vivo* 15,25 could be employed to resolve these issues.

## MECHANISMS OF ANTIPSYCHOTIC-MEDIATED MODULATION OF NEURONAL/BRAIN AUTOPHAGY

**Figure 1:**
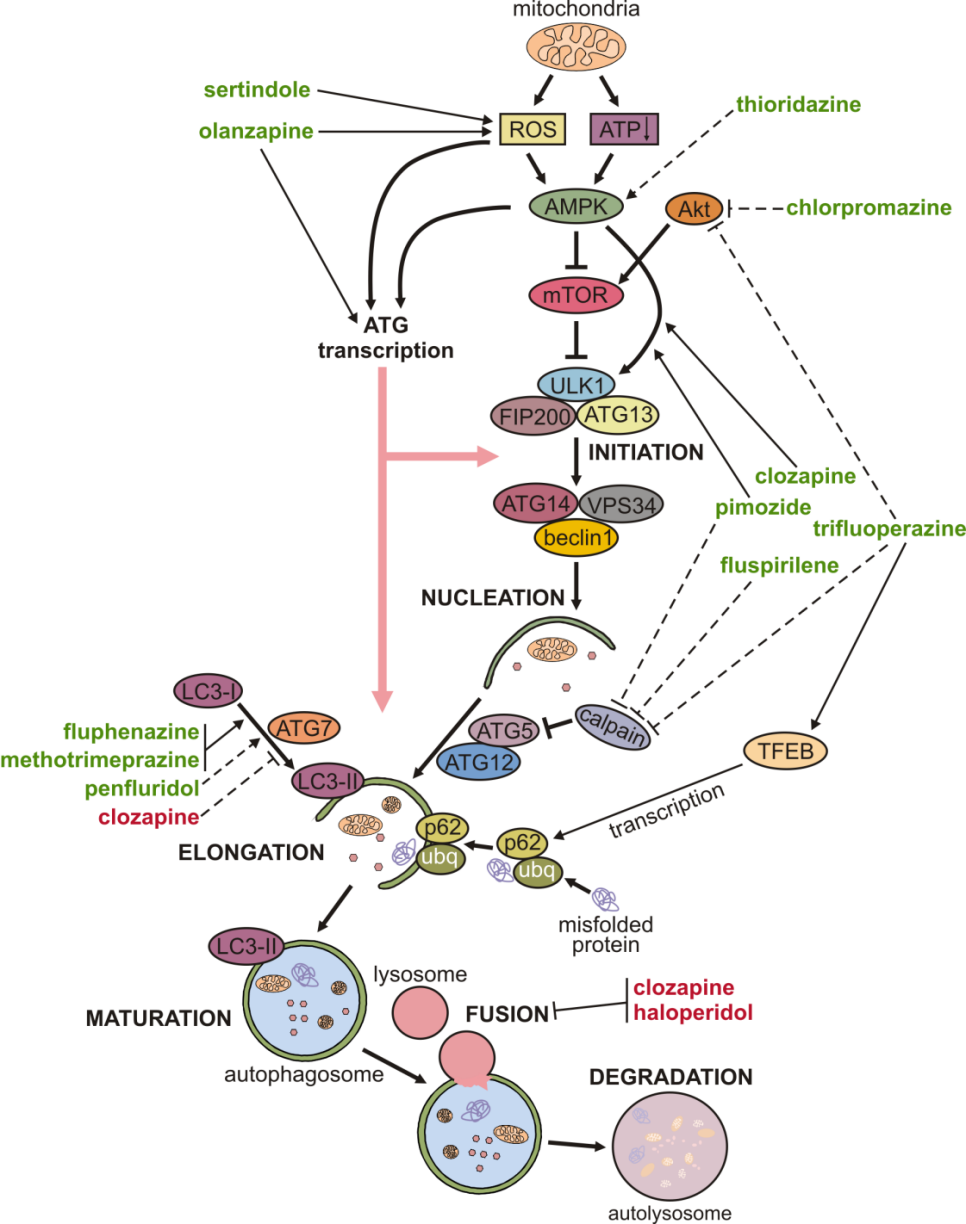
Figure 1: Mechanisms of autophagy regulation by antipsychotics. A simplified map of autophagy regulation is presented, including the sites of action of antipsychotic drugs shown as arrow-headed (activation/increase) or bar-headed lines (inhibition/decrease). The full and dashed lines refer to modulation in neurons/brain and non-neuronal cells, respectively. The drug names are marked green for autophagy stimulation, or red for autophagy inhibition. Some drugs are shown to directly modulate LC3 conversion only because no specific underlying mechanism was described. (ubq - ubiquitin).

The mechanisms underlying the modulation of neuronal autophagy by antipsychotics are delineated below and schematically depicted in **Fig. 1**. Autophagy is executed through hierarchical activation of autophagy-related (ATG) proteins organized in functional complexes that control different stages of autophagy process 26. Autophagosome biogenesis is initiated by the functional complex containing the mammalian ATG1 homologue Unc-51-like kinase (ULK)1, ATG13, and FIP200, which is recruited to the phagophore assembly site. Vesicle nucleation continues through the generation of phosphatidylinositol 3-phosphate, catalyzed by ULK1-activated complex containing the lipid kinase VPS34, beclin-1, and ATG14. The next step, vesicle elongation, is mediated by the two ubiquitin-like conjugation systems: ATG7 and ATG10 conjugate ATG5 to ATG12, and then ATG4, ATG7, and ATG3 conjugate phosphatidylethanolamine to LC3. LC3 (mammalian ATG8 homologue) is the signature autophagosome protein promoting the expansion of the autophagosomal membrane and its closure and fusion with the lysosome (maturation step), where the ubiquitinated cytoplasmic material bound to autophagic cargo receptor p62 is eventually degraded.

Autophagy is mainly regulated post-transcriptionally, through phosphorylation, ubiquitination, and/or acetylation of ATG proteins and their regulators, which alters their functional activity or changes their structure to modulate the affinity for binding partners 26. The initiation of autophagy is controlled by mammalian target of rapamycin (mTOR)-containing mTOR complex 1 (mTORC1), which inhibits autophagy by phosphorylating Unc-51-like autophagy activating kinase (ULK1, mammalian ATG1) 26. mTORC1 is activated by Akt and inhibited by AMP-activated protein kinase (AMPK), which also activates autophagy by directly activating ULK1 27. The induction of autophagy by olanzapine or pimozide was apparently independent of mTORC1 modulation, as neither drug was able to affect the phosphorylation of mTOR or mTORC1 substrate ribosomal p70S6 kinase 16,18. On the other hand, pimozide- and clozapine-triggered autophagy was associated with activation of AMPK and AMPK-dependent phosphorylation of ULK1 in SH-SY5Y neurons and in mouse hippocampus or rat frontal cortex *in vivo* 16,23. A genetic AMPK knockdown *in vitro*, and pharmacological AMPK suppression *in vivo*, reduced pimozide- or clozapine-triggered ULK1 phosphorylation and subsequent induction of autophagy 16,23, thus further supporting a role for AMPK-ULK1 interaction in the mTORC1-independent autophagy triggered by antipsychotics. AMPK-dependent autophagy is mainly activated by oxidative stress and the energy deficit reflected in the increase of AMP/ATP ratio, both resulting from mitochondrial dysfunction 27,28. Moreover, reactive oxygen species (ROS) can directly stimulate autophagy through redox modification of ATG proteins 29. Accordingly, sertindole-induced autophagy in SH-SY5Y neuronal cells was caused by ROS 17,18, while olanzapine-mediated mitochondrial depolarization led to oxidative stress-dependent autophagic clearance of damaged mitochondria in SH-SY5Y cells 18.

Autophagy is also regulated transcriptionally 26, and both AMPK and oxidative stress can stimulate transcription of ATG genes by activating various transcription factors such as transcription factor EB (TFEB), forkhead box O1/3, activating transcription factor 4, nuclear factor-κB, nuclear factor (erythroid-derived 2)-like 2, and p53 4,30. Transcriptional regulation of autophagy by antipsychotics is supported by the ability of olanzapine to increase the mRNA expression of ATG4, 5, 7, 12, and beclin-1 in SH-SY5Y neurons *in vitro* and in mouse brain *in vivo* 18. Similarly, trifluoperazine increased TFEB-dependent transcription of autophagic cargo receptor p62 in mitochondrially-stressed SH-SY5Y cells 19. Post-transcriptional regulation of autophagy by microRNA-mediated ATG mRNA cleavage and/or translational arrest has also been described 26. While there is presently no available direct evidence of post-transcriptional regulation of neuronal autophagy by antipsychotic drugs, it has been reported that penfluridol can downregulate the expression of microRNA-17 and -20a 31, which are shown to inhibit autophagic response by blocking the expression of ULK1 and ATG7, respectively 32,33.

The above delineated connections between antipsychotic-triggered neuronal autophagy and ROS, energy balance sensor AMPK, and stress-induced transcriptional activator TFEB indicate that autophagy is a part of the integrated stress response 34 of neurons to antipsychotic treatment. However, it remains on future studies to explore the exact connections between autophagy and other parts of the adaptive response to antipsychotics, as well as their role in neuronal homeostasis and function. Finally, it should be noted that some antipsychotics, such as haloperidol and clozapine, have been reported to suppress autophagy at the stage of autophagosome-lysosome fusion 24. It was proposed that haloperidol-mediated phosphorylation of the microtubule-associated protein tau and subsequent neurocytoskeletal disorganization 35 could impair autophagosome fusion with lysosomes, but this possibility was not experimentally tested.

### The role of dopaminergic/serotonergic blockade in autophagy modulation by antipsychotics

It has recently been shown that dopamine receptor subtypes differently regulate autophagy, with D2 and D3 (D2-like family) being positive, and D1 and D5 (D1-like family) negative regulators 36. The stimulation of D2 and D3 receptors was also able to induce beclin-1-dependent autophagy in neurons 37. Since most antipsychotic drugs are primarily antagonists of the D2-like family receptors, it seems unlikely that dopaminergic blockade was involved in their ability to induce autophagy. Accordingly, a phenoxazine-derived compound structurally related to phenotiazine antipsychotics, but lacking their antidopaminergic activity, readily induced neuronal autophagy 22, while D2 antagonist sulpiride failed to exert a similar effect 16. Also, the ability of both typical (fluphenazine, methotrimeprazine, pimozide, trifluoperazine) and atypical antipsychotics (olanzapine, sertindole) to stimulate neuronal autophagy argues against the involvement of serotonergic blockade in autophagy induction by the latter. Finally, as the inhibition of autophagic flux by haloperidol and clozapine was not mimicked by other antipsychotics, it is unlikely that it was mediated by dopaminergic/serotonergic blockade. It remains to be examined if chronic interference with dopaminergic/serotonergic signaling by antipsychotics might affect neuronal autophagy *in vivo*.

## CLINICAL SIGNIFICANCE OF NEURONAL AUTOPHAGY MODULATION BY ANTIPSYCHOTICS

The above findings indicate modulation of neuronal autophagy as a common denominator of intracellular action of antipsychotic drugs, raising the possibility that their therapeutic and/or side-effects might partly rely on this property. Indeed, a possible link between autophagy and schizophrenia has recently emerged, as the expression of autophagy-regulating genes, including autophagy-essential beclin-1, was found to be reduced in postmortem cortical and hippocampal tissues of schizophrenic individuals 38,39,40. Autophagy impairment might contribute to schizophrenia by interfering with the role of autophagy in synaptic plasticity 41 and removal of neurotoxic protein aggregates, such as those formed by the products of Disrupted-in-schizophrenia-1 gene 42. Accordingly, autophagy dysregulation has also been demonstrated in different animal models of schizophrenia 43,44,45 and pharmacological restoration of autophagic activity by microtubule assembly-promoting peptide davunetide was associated with behavioral improvements 44. It is therefore plausible that autophagy stimulation by antipsychotics such as trifluoperazine, fluphenazine, methotrimeprazine, olanzapine, pimozide, or sertindole might contribute to their beneficial effects by improving neuronal function.

Some studies suggest that long-term antipsychotic treatment may cause neurotoxicity and contribute to brain tissue volume loss associated with schizophrenia 46,47, but the underlying mechanisms are unknown. Autophagy activation by antipsychotic drugs has been shown to influence survival and death of neuronal cells in various experimental settings. The induction of autophagy by sertindole caused neuronal death *in vitro* 17, indicating a possible involvement of autophagy in the neurotoxicity of antipsychotics. On the other hand, olanzapine-mediated autophagy was associated with oxidative stress and mitochondrial dysfunction in the absence of overt neurotoxicity 18. Moreover, pharmacological and/or genetic inhibition of olanzapine-triggered autophagy led to impaired clearance of damaged mitochondria and apoptotic neuronal death both *in vitro* and *in vivo*, thus unmasking the neurotoxic action of the drug 18. These data suggest that the neurotoxicity of antipsychotics might be increased in patients with impaired autophagy due to aging, neuroinflamation, and/or neurodegeneration 48.

Understanding the possible involvement of antipsychotics in neurodegeneration is even more important having in mind their increasing use in the treatment of psychotic or related behavioral disturbances in patients with neurodegenerative diseases 49. Interestingly, a line of recent evidence indicates that autophagy induction by antipsychotics might actually have beneficial effects on mitochondrial dysfunction and proteotoxic aggregation in these disorders. Trifluoperazine-induced autophagy rescued human dopaminergic neurons from the toxic effects of α-synuclein accumulation *in vitro* 21, and reduced dopaminergic neuron loss in a zebrafish model of Parkinson’s disease 19. Autophagy stimulation by fluphenazine and methotrimeprazine enhanced survival of primary rat neurons and human stem cell-derived neurons by clearing mutant TAR DNA-binding protein 43 in *the in vitro* model of amiotrophic lateral sclerosis 20. Pimozide-triggered autophagy decreased abnormally phosphorylated tau aggregates in neuronal cells *in vitro* and *in vivo*, leading to an improvement of memory deficit in a mouse model of Alzheimer’s disease 16. Similarly, haloperidol protected striatal neurons from dysfunction induced by accumulation of mutated huntingtin *in vivo*, although the role of autophagy in this effect was not investigated 50.

The above findings provide a proof of concept for using autophagy-activating drugs to improve clearance of proteotoxic aggregates in neurodegenerative diseases. Moreover, as mTORC1 inhibition in neurons does not always trigger autophagic response 51, mTORC1-independent autophagy induction by antipsychotics might be a more reliable therapeutic option than the use of classic mTORC1-inhibiting drugs like rapamycin and its analogues. Such an approach would ideally require elimination of the antidopaminergic activity and the related side-effects that could compromise the use of antipsychotics in neurodegenerative disorders. On the other hand, treatment with antipsychotics was associated with a significant increase in neurofibrillary tangles and amyloid plaques in the frontal lobe cortex of patients suffering from dementia 52. Therefore, the possibility that some antipsychotics, as shown for haloperidol and clozapine, might block autophagic proteolysis 24, thus potentially contributing to accumulation of proteotoxins and neurodegeneration, must be carefully considered.

## ANTIPSYCHOTIC-MEDIATED MODULATION OF AUTOPHAGY IN NON-NEURONAL CELLS

In addition to modulating autophagy in neurons, antipsychotics have been shown to affect autophagy induction in other cell types (**Table 2**) through a variety of mechanisms (**Fig. 1**). Similar to findings obtained in neurons, antipsychotics mostly stimulated autophagy in non-neuonal cells, but inhibitory effects were also observed.

**Table 2:** Table 2. Modulation of autophagy by antipsychotics in non-neuronal cells. "Flux" refers to autophagic flux; ↑ denotes increase/activation; ↓ denotes decrease/inhibition; n.a. - not assessed; ATZ - α1-antitrypsin Z; ER - endoplasmic reticulum; PKC - protein kinase C.

**Agent**	**Cells/Tissues**	**LC3-II**	**Flux**	**Mechanisms**	**Effects**	**Ref.**
Thioridazine	GBM8401, U87MG glioma cells	↑	↑	AMPK↑	Apoptotic death; Tumor growth in vivo↓	53
Trifluoperazine	MDA-MB-231 breast cancer cells	↑	n.a.	n.a.	Migration↓; Invasiveness↓	54
Trifluoperazine	Ca922, SCC2095, primary oral cancer cells	↑	n.a.	Akt/mTORC1↓	Apoptotic death; Tumor growth in vivo↓	58
Trifluoperazine	HeLa cells	↑	↑	n.a.	Bacterial clearance	61
Trifluoperazine	H4 glioma cells	↑	↑	Ca^2+^↓, calpain↓, ATG5 cleavage↓	Clearance of mutant; poliglutamine	13,59
Fluspirilene	H4 glioma cells	↑	↑	Ca^2+^↓, calpain↓, ATG5 cleavage↓	Clearance of mutant; poliglutamine	13,59
Pimozide	H4 glioma cells	↑	↑	Ca^2+^↓, calpain↓, ATG5 cleavage↓	Clearance of mutant; poliglutamine	13,59
Penfluridol	Panc-1, AsPC-1, BxPC-3; pancreatic cancer cells	↑	↑	n.a.	Apoptotic death; Tumor growth in vivo↓	55
Chlorpromazine	U87MG glioma cells	↑	↑	Akt/mTORC1↓	Apoptotic death; Tumor growth in vivo↓	56
Olanzapine	LN229, T98 glioma cells	↑	↑	n.a.	Apoptotic death; Tumor growth in vivo↓	57
Clozapine	A549, H1299 lung cancer cells	↑	↑	n.a.	Cell cycle arrest; Cell death	60
Clozapine	Mouse skeletal muscle	↓	n.a.	PKCβ↑, LC3 conversion↓	Lipid droplet clearance↓; Weight gain	63
Fluphenazine	Mouse liver	↑	n.a.	n.a.	ATZ clearance; Liver fibrosis↓	62

### Autophagy induction by antipsychotics in non-neuronal cells

Phenothiazine antipsychotics trifluoperazine, thioridazine, and chlorpromazine, as well as fluspirilene, pimozide, penfluridol, olanzapine, and clozapine all induced autophagy in various cancer cell types, including glioma, breast, lung, pancreatic, and oral cancer cells 13,53,54,55,56,57,58,59,60. Autophagy induction by antipsychotics reduced migration and invasiveness of cancer cells 54, or cause their apoptotic/autophagic death *in vitro* and *in vivo* 53,55,56,57,58,59,60. Moreover, trifluoperazine was used as a lead compound to develop autophagy-inducing anticancer agents with improved efficacy and reduced toxicity, thus providing a proof-of-concept for designing phenothiazine-derived anticancer drugs 58. Trifluoperazine, fluspirilene, and pimozide increased autophagic clearance of mutant poliglutamine in H4 glial cells 13, trifluoperazine-induced autophagy promoted bacterial clearance in HeLa cells 61, and fluphenazine-triggered autophagy reduced the accumulation of mutant α1-antitrypsin in hepatocytes and subsequent liver fibrosis 62. Chlorpromazine- and trifluoperazine-mediated autophagy in cancer cells was dependent on Akt inhibition-mediated mTORC1 suppression 56,58, while mTORC1-unrelated AMPK activation was apparently responsible for thioridazine-induced autophagy 53. Fluspirilene, pimozide, and trifluoperazine stimulated autophagy by inhibiting Ca^2+^-dependent cleavage of ATG5 by calpain, which in turn increased the levels of ATG5-ATG12 conjugate required for autophagosome formation 59. Moreover, a structure-activity relationship study of 83 fluspirilene derivatives demonstrated that only those inhibiting Ca^2+^ channels and subsequent calpain-mediated ATG5 cleavage were also able to induce autophagy 59, suggesting a critical role of this mechanism in autophagy induction by antipsychotics.

### Autophagy inhibition by antipsychotics in non-neuronal cells

In contrast to the above data, clozapine-mediated protein kinase Cβ activation inhibited LC3 conversion and autophagic clearance of lipid droplets in the mouse skeletal muscle *in vivo* 63, thus providing a novel insight into the mechanisms underlying the clozapine-induced weight gain. Also, N-n-butyl haloperidol iodide protected cardiomyocytes from hypoxia/reoxygenation injury by reducing beclin-1 and ATG5 levels and inhibiting autophagy 64, but it was not assessed whether these effects were actually due to the chemical modification of the parental compound.

While it would be interesting to compare the mechanisms of antipsychotic-mediated autophagy modulation in neurons and non-neuronal cells, the number of studies providing a mechanistic insight required for such a comparison is presently rather limited. Nevertheless, it appears that autophagy induction by antipsychotics in non-neuronal cells might more often rely on mTORC1 inhibition, which is consistent with the relative insensitivity of neuronal autophagy to mTORC1 suppression 51. Also, having in mind the critical role of calcium signaling in neuronal function 65, it would be important to investigate if the inhibition of Ca^2+^ channels by antipsychotics, causing autophagy in cancer cells 59, might affect autophagy in neurons as well. Further studies are required to directly compare signaling events involved in autophagy modulation by antipsychotics in neurons and other cell types, thus facilitating the development of selective modulators of neuronal autophagy.

## CONCLUSIONS

The present review demonstrates that many typical (trifluoperazine, fluphenazine, fluspirilene, chlorpromazine, methotrimeprazine, thioridazine, pimozide, penfluridol) and atypical antipsychotics (olanzapine, sertindole) induce autophagy in neuronal or other cell types, mainly through AMPK-mediated mTORC1-independent or mTORC1-dependent mechanisms, respectively. On the other hand, antipsychotics such as clozapine and haloperidol in certain conditions caused inhibition of autophagic flux, the mechanisms and significance of which remain to be explored. The induction of autophagic response by antipsychotic drugs might contribute to reducing neuronal dysfunction in schizophrenia, but also to the adverse effects associated with their long-term use, including brain volume loss and weight gain. If antipsychotics, in accordance with their ability to induce autophagy in glioma cells, could influence normal glia in a similar way, this might have further implications for schizophrenia therapy. Moreover, having in mind the increasing use of antipsychotics in disorders other than schizophrenia, understanding the effects that autophagy activation by these drugs might have on the brain has important clinical implications beyond schizophrenia treatment. This seems particularly important in neurodegenerative diseases, where mTORC1-independent autophagy induction by antipsychotic derivatives devoid of anti-dopaminergic activity might help to increase the clearance and reduce neurotoxicity of aggregated proteotoxins. Moreover, the ability of different antipsychotics to preferentially cross the blood-brain barrier in certain brain areas 66 could be exploited to enable brain region-selective autophagy activation. Finally, the anticancer effects of autophagy induction by antipsychotics make plausible their repurposing as adjuncts to standard cancer therapy. A further in-depth investigation of the mechanisms and effects of autophagy triggered in neuronal and other cells/tissues by antipsychotics is required for understanding the biological actions, increasing the beneficial/side-effect ratio, and broadening the therapeutic scope of this important class of drugs.

## Abbreviations

AMPK: AMP-activated protein kinase
ATG: autophagy-related
LC3: light chain
mTOR: mammalian target of rapamycin
ROS: reactive oxygen species
TFEB: transcription factor EB
ULK: Unc-51-like autophagy activating kinase
